# The Huxley Lecture

**Published:** 1906-10-06

**Authors:** 


### The Opening of the Medical Schools.

### THE HUXLEY LECTURE.

### PROFESSOR PAWLOW'S ADDRESS AT CHARING CROSS HOSPITAL.

New Physiological Methods.

The Huxley Lecture was delivered at the Charing Cross Hospital by Professor Ivan P. Pawlow, Professor of Physiology in the University of St. Petersburg. He was introduced by Lord Kilmorey, the chairman of the Hospital Board, and delivered his address in German on the subject of the experimental facts recently added to our knowledge on the reflex excitability of the secretion of saliva and its bearing on our knowledge of the operation of the stimulus in the central nervous system. The interesting lecture bore closely on the old statement as to the close connection that exists between the advance of science and the advance of method at the disposal of that science. When questions have to be put to the living animal or plant it is of all things necessary that the reaction shall be conveyed only by those stimuli which ought to be applied, and that there should be no extraneous stimuli or circumstances interfering with the response. An enormous advance was made in this direction by the introduction of anæsthetics and the abolition of pain from physiological laboratories. But the physiological conditions of the organisms are not normal under anæsthesia—they are poisoned by the anæsthetic. It has been Professor Pawlow's great service to medical science generally that he has taught how to experiment on living animals in perfect physiological conditions without anæsthetics, pain, or even discomfort. Unsurpassed surgical skill has succeeded in producing anatomical abnormalities which were not attended with pain or disturbance of the physiological functions of the animal. A new gospel of physiology has been supplied by Pawlow. He has shown processes of the digestion and assimilation of food, and in his lecture he has shown a still more daring step. It was supposed that investigation of the higher functions of animals and of man should be left to the pathologist or the psychologists who have studied them by the subject methods. Pawlow gives us purely objective methods, from which progress will follow and bring as great benefit to medicine as did his researches on digestion.

For a consistent investigator there is in the higher animals only one thing to be considered—that of the other external response of the animal to external impressions. This response may be extremely complicated in comparison with the reaction of animals of a lower class and infinitely complicated in comparison with the response of any inanimate object that could be named, but the principle involved in the question remains the same. Strictly speaking, natural science is under an obligation to determine only the precise connection which exists between the given natural phenomenon and the responsive faculty of the living organism with respect to this phenomenon—or, in other words, to ascertain completely how the given living object maintains itself in constant relation with its environment. The question is simply whether this law is now applicable to the examination of the higher functions of the higher animals. A serious endeavour to institute inquiries in this direction is the only reasonable answer to this question. The only response of the animals to external impressions was a physiologically unimportant process—namely, the excretion of saliva. The experimenter always used perfectly normal animals. By means of a systematic procedure easy of manipulation it was possible to obtain an exact observation of the work of the salivary glands at any desired time. It is already well known that there is always a flow of saliva in the dog when something to eat is given to it or when anything is forcibly introduced into its mouth. Here we have a well-known physiological process—reflex action. Reflex action is the response of the animal to external influences accomplished by the aid of the nervous system.

The centripetal paths are always stimulated primarily and the centrifugal paths secondarily with the interposition of the central nervous system.

In the first place they arise from all the bodily surfaces which are sensitive to stimulation, even from such regions as the eye and the ear, from which an ordinary reflex action affecting the salivary glands is never known to proceed. It must be observed that ordinary salivary reflexes may originate not only from the cavity of the mouth but also from the skin and the nasal cavity; the skin, however, produces this effect only when it is subjected to some destructive process such as cutting or erosion by caustics, while the nasal cavity produces this effect only through the contact of vapours or gases, such as ammonia, which cause local irritation, but never through the agency of odours properly so-called. In the second place, a conspicuous feature of these reflexes is that they are in the highest degree inconstant. All stimuli introduced into the mouth of the dog unfailingly give a positive result in reference to the secretion of saliva, but the same objects when presented to the eye, the ear, etc., may be sometimes efficient and sometimes not. In consequence only of the last-mentioned fact the new reflexes have been termed "conditioned reflexes," and for the sake of distinction the old ones are "unconditioned."

Every conditioned stimulus becomes totally ineffective on repetition, the explanation being that the reflex action ceases. But there is a way in which the reflex may be restored immediately. All that is necessary is to obtain a repetition of the unconditioned reflex, as, for instance, by pouring something acid into the dog's mouth and then either showing it to him or letting him smell it. Concluding, he said there is to be seen an essential property of these manifestations which they have in common with the former ones—that is, the existence of a very sensitive point in the central nervous system, and in consequence of its position this point becomes the destination of all the important stimuli coming from the external world to make impressions on the receptive cells of the higher regions of the brain.

These manifestations present great facilities for exact investigation, being in this respect scarcely inferior to the ordinary physiological manifestations. I am here referring to the ease with which they may be repeated, to their character of uniformity under similar conditions of environment, and to the fact that they are capable of further subdivision experimentally. It is inevitable that opinions formed on this subject must be objective only. The introduction of a few subjective considerations which we admitted now and again for the purpose of comparison seemed on further reflection to be an act of violence or an affront.

Up to the present time the physiology of the eye, the ear, and other superficial organs which are of importance as recipients of impressions, has been regarded almost exclusively in its subjective aspect; this presented some advantages, but at the same time, of course, limited the range of inquiry. In the investigation of the conditioned stimuli in the higher animals this limitation is got rid of and a number of important questions in this field of research can be at once examined with the aid of all the immense recources which experiments on animals place in the hand of the physiologist. The investigation of the conditioned reflexes is of very great importance for the physiology of the higher parts of the central nervous system. Hitherto this department of physiology has throughout most of its extent availed itself of ideas not its own, but ideas borrowed from psychology, from which it is now likely to be liberated. The conditioned reflexes lead us to the consideration of the position of animals in nature; this is a subject of immense extent and one that must be treated objectively. The physiologist can and must examine this question in connection with progressive and systematic derangement of the central nervous system in order that he may ultimately arrive at an exact knowledge of the mechanism involved.

It is plain that this conquest which physiology has still to make consists for the most part of the solution of questions which hitherto vex and perplex humanity. Men will possess incalculable advantages and an extraordinary power over themselves when scientific investigators will subject other men to the same external analysis as they would employ for any natural object, and when the human mind will contemplate itself not from within but from the outside.

